# Influences of mesoporous magnesium calcium silicate on mineralization, degradability, cell responses, curcumin release from macro-mesoporous scaffolds of gliadin based biocomposites

**DOI:** 10.1038/s41598-017-18660-9

**Published:** 2018-01-09

**Authors:** Sicheng Wang, Zhengrong Gu, Zhiwei Wang, Xiao Chen, Liehu Cao, Liang Cai, Quan Li, Jie Wei, Jung-Woog Shin, Jiacan Su

**Affiliations:** 1 Department of Trauma Orthopaedics, Changhai Hospital, Second Military Medical University, Shanghai, 200433 China; 2 Department of Orthopaedics, Zhongye Hospital, Shanghai, 200941 China; 3 The Department of Orthopaedics, Jing’an District Centre Hospital of Shanghai (Huashan Hospital, Fudan University Jing’An Branch), Shanghai, 200040 China; 4 Key Laboratory for Ultrafine Materials of Ministry of Education, East China University of Science and Technology, Shanghai, 200237 China; 5 Department of Biomedical Engineering, Inje University, Gimhae, 621749 Republic of Korea

## Abstract

Macro-mesoporous scaffolds based on wheat gliadin (WG)/mesoporous magnesium calcium silicate (m-MCS) biocomposites (WMC) were developed for bone tissue regeneration. The increasing amount of m-MCS significantly improved the mesoporosity and water absorption of WMC scaffolds while slightly decreased their compressive strength. With the increase of m-MCS content, the degradability of WMC scaffolds was obviously enhanced, and the decrease of pH value could be slow down after soaking in Tris-HCl solution for different time. Moreover, the apatite mineralization ability of the WMC scaffolds in simulated body fluid (SBF) was obviously improved with the increase of m-MCS content, indicating good bioactivity. The macro-mesoporous WMC scaffolds containing m-MCS significantly stimulated attachment, proliferation and differentiation of MC3T3-E1 cells, indicating cytocompatibility. The WMC scaffold containing 40 w% m-MCS (WMC40) possessed the highest porosity (including macroporosity and mesoporosity), which loaded the highest amount of curcumin (CU) as well as displayed the slow release of CU. The results suggested that the incorporation of m-MCS into WG produced biocomposite scaffolds with macro-mesoporosity, which significantly improved water absorption, degradability, bioactivity, cells responses and load/sustained release of curcumin.

## Introduction

The applications of mesoporous biomaterials for bone tissue regeneration has been drawn more and more attentions over the past decades, such as silicon oxide, calcium silicate, bioglass, etc^1,2^. These mesoporous biomaterials with large specific surface area and high pore volume have been demonstrated to possess enhanced biological performances (such as bioactivity, degradability, drug load/release, etc.)^2,3^. Moreover, the mesoporous biomaterials significantly promoted attachment, proliferation and differentiation of bone marrow stem cells/osteoblasts, and improve new bone tissue regeneration^4^.

Recently, natural biopolymers (such as plant protein, starch, etc.) got from the agricultural products, have attracted much attentions as the biodegradable polymer materials and were widely used in the field of regenerative medicine, such as bone repair materials and implantable cardiovascular devices, due to their good biocompatibility and biodegradability^5,6^. Among the natural biopolymers, the wheat protein (one of the plant proteins), which derived from the cereal crop, is one of the most promising biodegradable polymers for regenerative medicine applications. Wheat protein subunits are linked together by disulfide and hydrogen bonds between cysteine residues to form the very large protein polymers^6^. Moreover, the types of amino acid in wheat protein are relatively abundant, which can provide enough nutrients for tissue regeneration^7^.

Mesoporous bioglasses (MBGs) have recently shown promise as bone reconstructed biomaterials^8^. MBGs can load osteogenic agents and drugs, which promote the growth of new bone^9^. As one of the MBGs, mesoporous magnesium calcium silicate (m-MCS) bioglass with large surface area and high pore volume exhibited rapid degradability, good *in vitro* bioactivity and cytocompatibility^10^. Furthermore, the degradable products of m-MCS cause weak alkaline microenvironment, which is favorable for cell growth and tissue regeneration^11^. However, like other inorganic scaffolds, m-MCS is very brittle and lacks antibacterial activity. This limits its applications in the treatment of bone defects, especially large bone defects complicated by infection.

Infection is one of the most serious and devastating complications faced by the millions of patients who undergo orthopedic procedures annually^12^. It increases the incidence of osteomyelitis, bone necrosis, septicemia, and loss of mobility in the dead zone^13^. Therefore, more effective antimicrobial agents suitable for use in prophylaxis and treatment should be developed, especially agents effective against antibiotic-resistant organisms. Curcumin (CU) is a biologically active substance found in the roots of *Curcuma longa* plant^14^. CU is a low molecular weight natural yellow-orange polyphenol compound, which has been used in wound healing, diabetes and cardiovascular ailments owing to its remarkable antibacterial, anti-inflammatory and antitumor activity^15,16^. Therefore, in this study, the macro-mesoporous scaffolds containing wheat gliadin (WG) and m-MCS composites (WMC) were developed by incorporating m-MCS into WG, which was crosslink with genipin. The aim of this study is to investigate the effects of m-MCS on porosity, water absorption, apatite mineralization, degradability, cell responses, CU load/release and antibacterial property of macro-mesoporous scaffolds for bone tissue engineering application.

## Results

### Characterization of scaffolds

**Figure 1:**
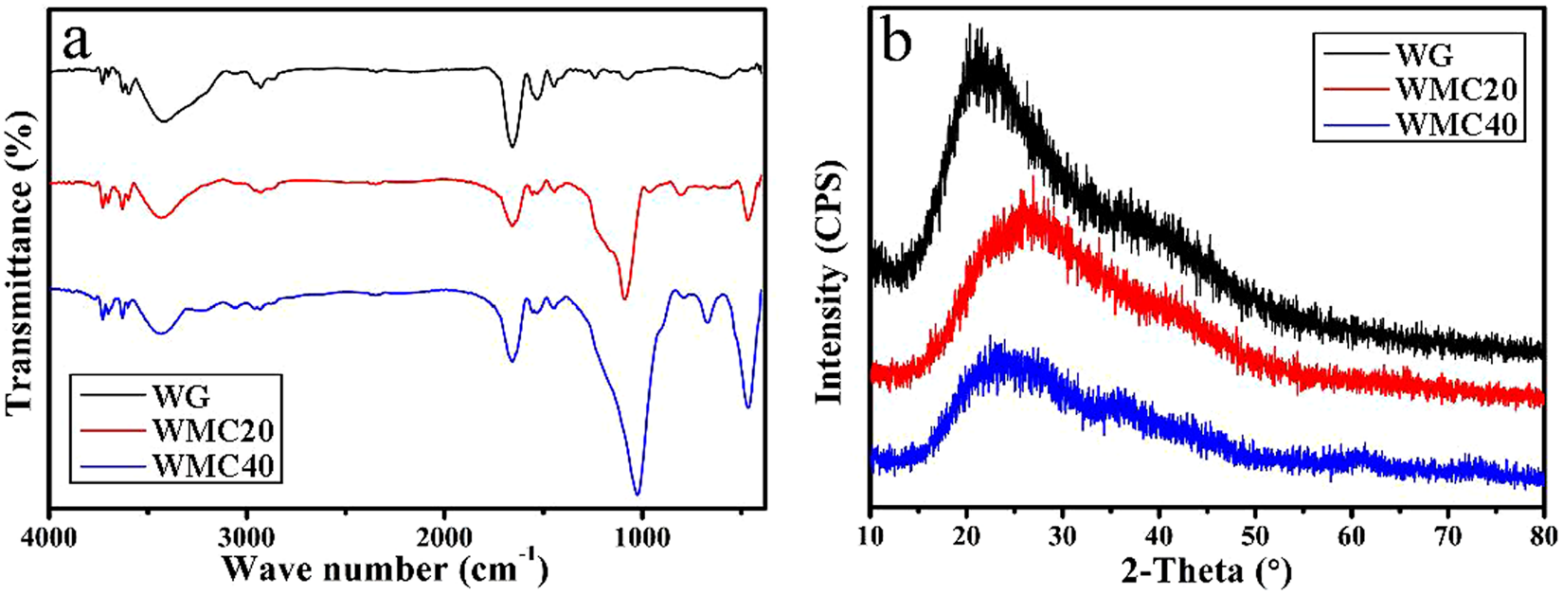
FTIR (**a**) and XRD (**b**) of WG, WMC20 and WMC40 scaffolds.

Figure 1a shows the fourier transform infrared spectroscopy (FTIR) of WG, WMC20 and WMC40 scaffolds. The peak at 1656 cm^−1^ was assigned to the amide bands, indicating that the carboxymethyl group of genipin has reacted with amino group to form secondary amide. The broad peaks at 3100–3400 cm^−1^ revealed the presence of N-H stretching of amide band, whereas the peaks at 1529 cm^−1^ (N-H bending vibration) and 1445 cm^−1^ (C-N stretching vibration) were ascribed to CO-NH amide bond, which was generated by genipin crosslinking WG^17^. The peaks at 1080 cm^−1^ and 500 cm^−1^ were found in both WMC20 and WMC40, which were attributed to Si-O-Si stretching, indicating the presence of m-MCS^18^. Figure 1b presents the X-ray diffraction (XRD) of WG, WMC20 and WMC40 scaffolds. It was found that no crystalline phase was found in the three kinds of scaffolds. The WG crosslinking with genipin showed an amorphous structure, and WMC20 and WMC40 with m-MCS also showed the amorphous structure.

**Figure 2:**
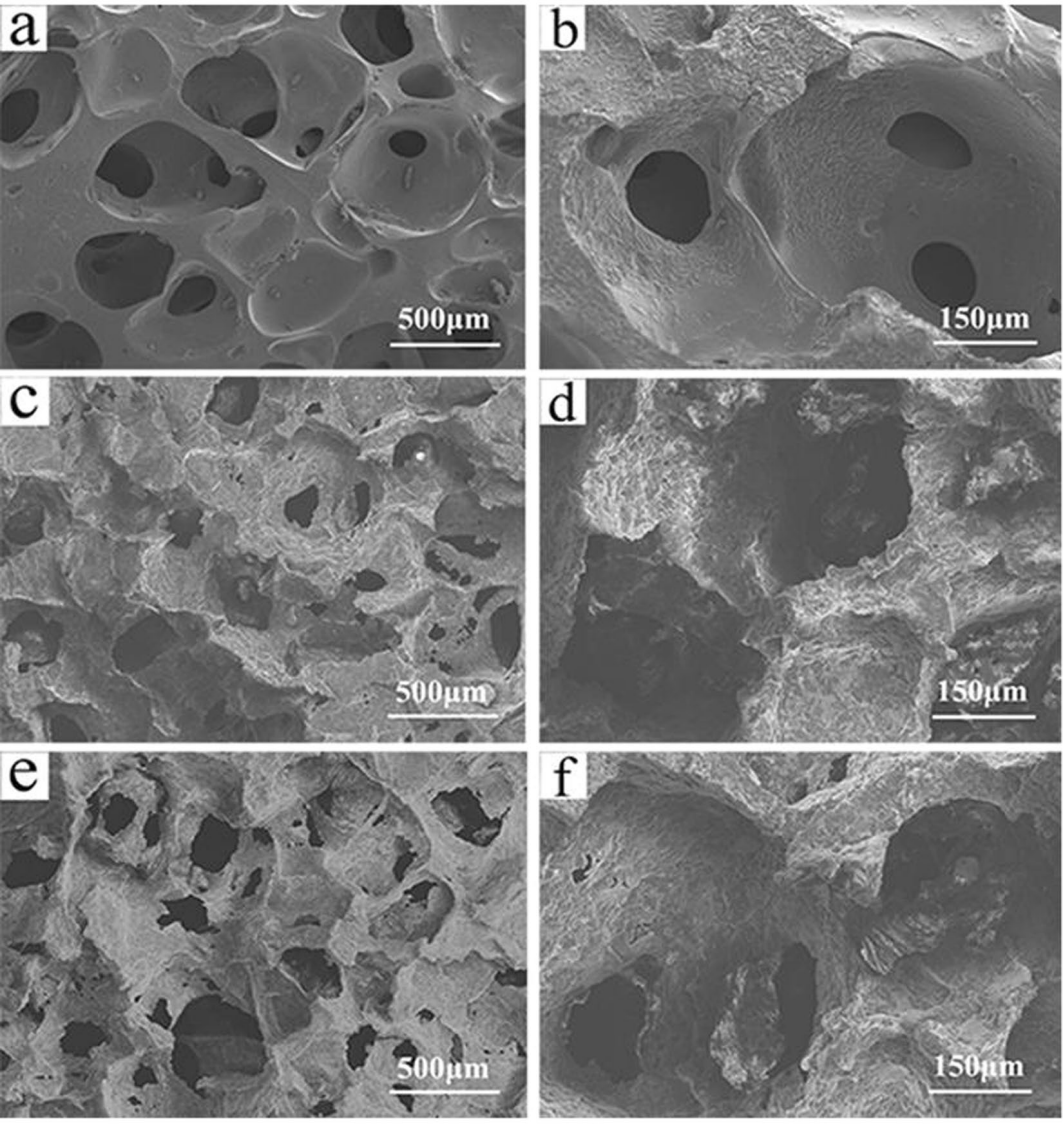
SEM images of surface morphology of WG (**a**,**b**), WMC20 (**c**,**d**) and WMC40 (**e**,**f**).

Figure 2 shows the scanning electron microscopy (SEM) images of the surface morphology and microstructure of the WG, WMC20 and WMC40 scaffolds. The scaffolds showed a structure of interconnected macropores with the pore size of about 500 μm. The surface of WG was smooth while the WMC20 and WMC40 became coarse with the increase of m-MCS content in the composites. From the TEM image of as-prepared m-MCS (Supplementary Figure 1), the distinct ordered mesopores could be observed inside m-MCS, indicating the potential of load drug (curcumin).

### Porosity and compressive strength, water absorption of scaffolds

Table 1 reports the porosity of the scaffolds. It was found that the porosity of the WG, WMC20 and WMC40 scaffolds was 53.4%, 70.1% and 77.2%, respectively. In addition, the compressive strengths of the WG, WMC20 and WMC40 scaffolds were 1.93, 1.86, and 1.71, respectively (Supplementary Figure 3).

**Table 1:** Porosity and compressive strength of scaffolds.

Samples	Porosity (%)	Compressive strength (MPa)
WG	53.4 ± 3.1%	1.93 ± 0.05
WMC20	70.1 ± 5.3%	1.86 ± 0.03
WMC40	77.2 ± 3.4%	1.71 ± 0.03

**Figure 3:**
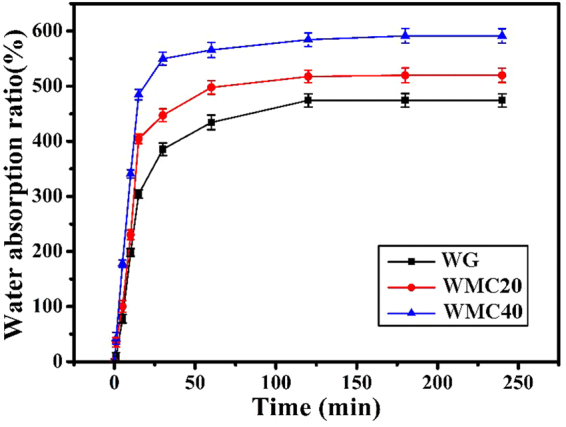
Water absorption of WG, WMC20 and WMC40 scaffolds.

Figure 3 reveals the water absorption of the WG, WMC20 and WMC40 scaffolds. The water absorption of the scaffolds significantly increased with the increase of m-MCS content. After incubated in de-ionized water for 240 min, the water absorption of WMC40 scaffolds was 591.1% while WG and WMC20 was 474.2% and 519.6%, respectively.

### Degradability of scaffolds in Tris-HCl solution

**Figure 4:**
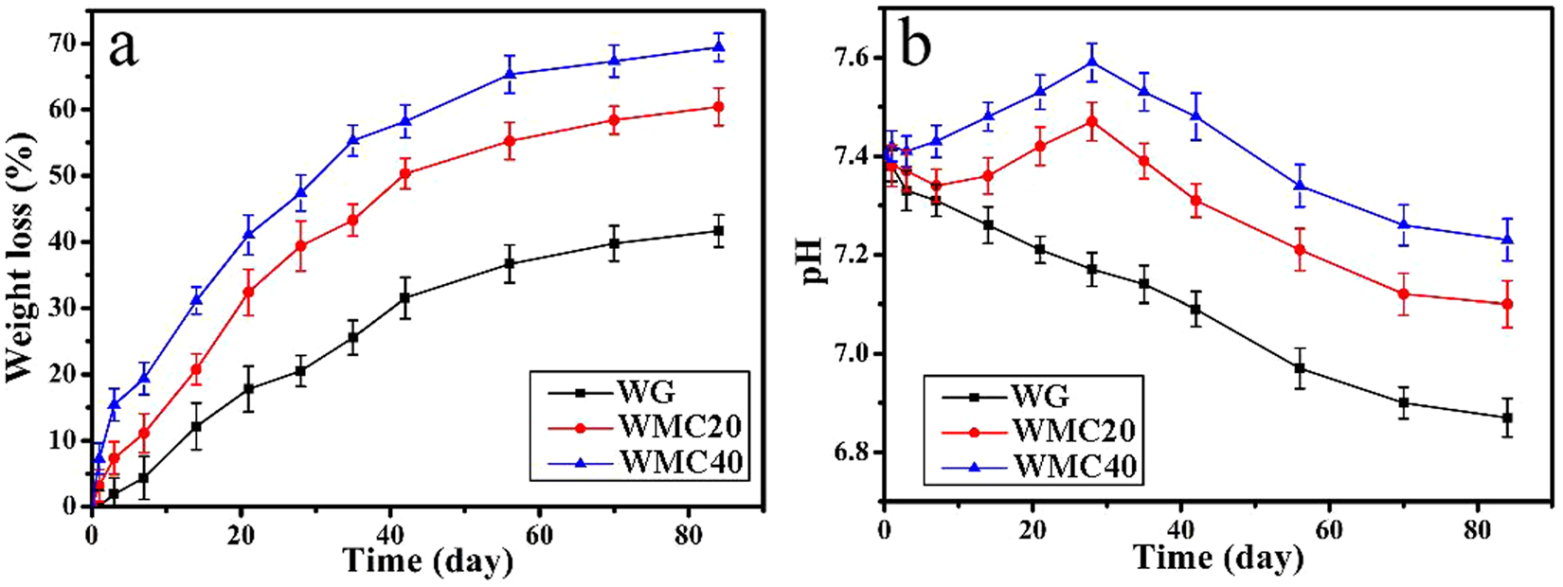
Weight loss (**a**) of the scaffolds and change of pH (**b**) in solution after WG, WMC20 and WMC40 scaffolds soaked into Tris-HCl solution with time.

The degradability of the scaffolds was determined by monitoring the weight loss of the scaffolds after soaked into in Tris-HCl solution for different time. Figure 4a presents the weight loss of the scaffolds soaking into Tris-HCl solution with time. After soaked for 84 days, the weight loss of WG, WMC20 and WMC40 scaffolds were 41.7%, 60.4% and 69.5%, respectively. Figure 4b exhibits the change of pH value of the solution after the scaffolds soaking into Tris-HCl solution with time. The pH of the solution for WG decreased from 7.40 to 6.87 after 84-day immersion. However, after 28-day immersion, the pH for WMC40 slightly increased from 7.40 to 7.59, and for WMC20 increased from 7.40 to 7.47. Additionally, after 84-day immersion, the pH for WMC40 decreased to 7.23 while WMC20 declined to 7.10.

### Mineralization of scaffolds in SBF

**Figure 5:**
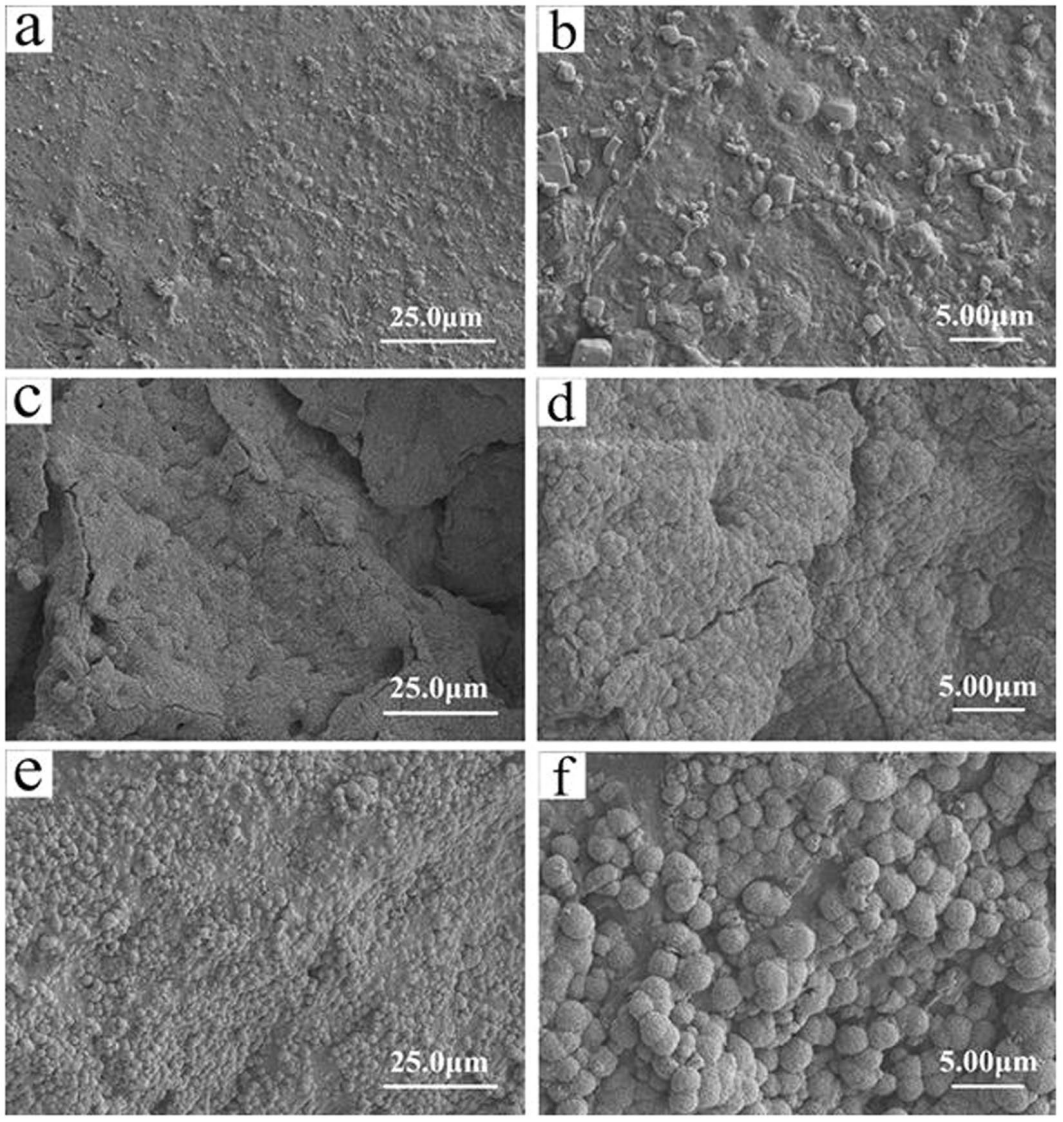
SEM images of surface morphology of mineralization of WG (**a**,**b**), WMC20 (**c**,**d**) and WMC40 (**e**,**f**) scaffolds after immersing in SBF for 7 days.

Figure 5 shows SEM images of surface morphology of the scaffolds after soaking into in SBF for 7 days. A small amount of irregular particles were found on the surface of WG (Fig. 5a,b). In contrast, the surface of WMC20 was covered with deposits layers, suggesting apatite mineralization (Fig. 5c,d). In addition, compared with WMC20, much denser deposits layers were found on the surface of WMC40, which were apatite microspheres (Fig. 5e,f).

**Figure 6:**
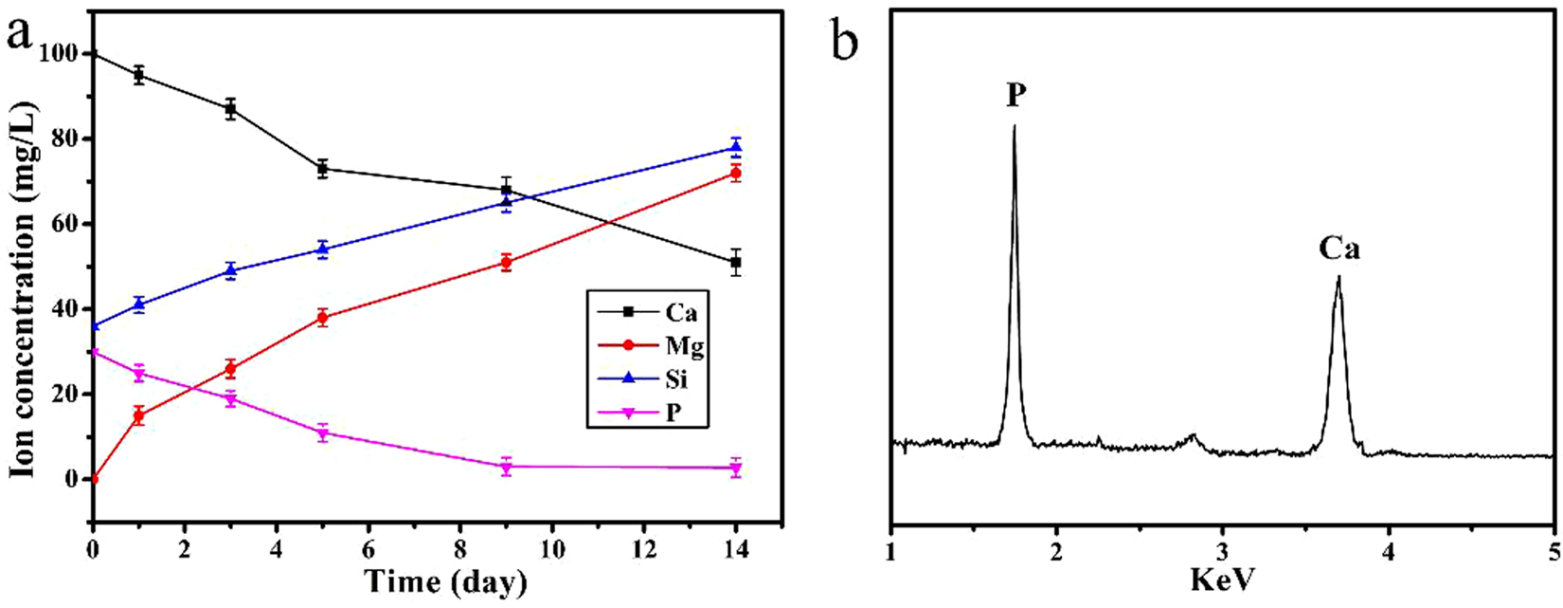
Changes of ions concentrations in SBF solution (**a**) after WMC40 immersion for different periods, and EDS (**b**) of WMC40 after soaking in SBF for 7 days.

Figure 6a reveals the changes of ions concentrations (Ca, Mg, Si and P) in SBF solution after WMC40 immersion for different periods (1, 3, 5, 7 and 14 days). Mg and Si ions exhibited an increase trend while Ca and P ions showed significantly decline. Figure 6b shows the energy dispersive spectroscopy (EDS) of WMC40 after soaking in SBF for 7 days. It revealed that Ca and P peaks were found, and the Ca/P ratio of the surface of the WMC40 scaffold was 1.60, which was close to that of stoichiometric hydroxyapatite (Ca/P: 1.67)^19^. From the results of FTIR analysis (Supplementary Figure 2), the characteristic peaks at 565 cm^−1^ and 604 cm^−1^ were corresponded to the bending vibration of P-O, and characteristic peak at 1045 cm^−1^ was corresponded to the stretching vibration of P-O, which further confirmed the formation of apatite on the scaffolds.

### Cell morphology on scaffolds

**Figure 7:**
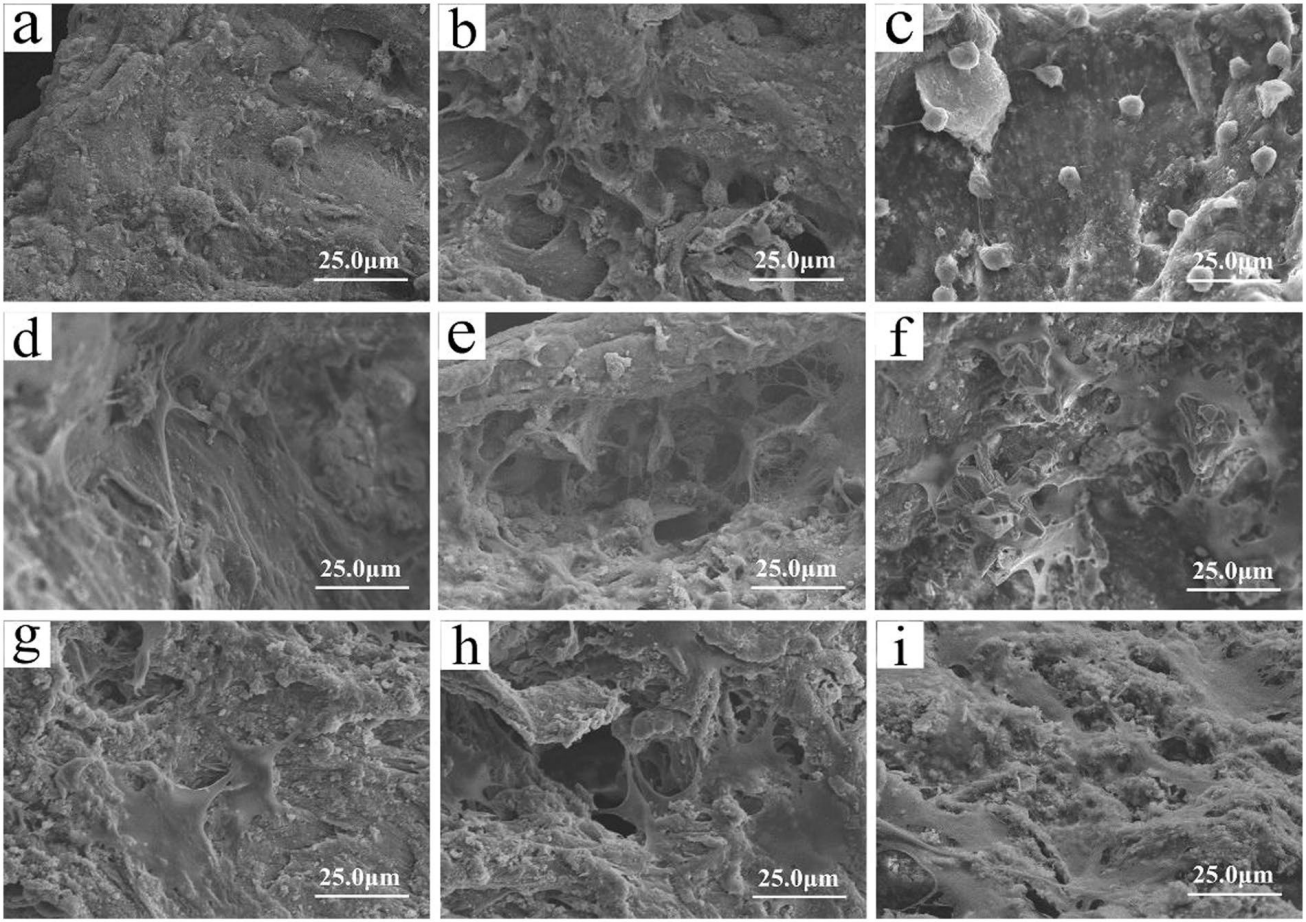
SEM images of cell morphology on WG (**a**,**d**,**g**), WMC20 (**b**,**e**,**h**) and WMC40 (**c**,**f**,**i**) scaffolds after being cultured for 1 (**a**,**b**,**c**), 3 (**d**,**e**,**f**) and 5 (**g**,**h**,**i**) days.

Figure 7 exhibits the SEM images of cell morphology on the scaffolds after being cultured for different time. At day 1, the morphology of cells attached on WG, WMC20 and WMC40 was round, and no contact with each other of the cells was found (Fig. 7a–c). At day 3, the spherical cells shifted to fusiform with parapodiums attaching on the walls of scaffolds, and the cells gradually formed groups on WMC20 and WMC40 (Fig. 7e,f) while the cells on WG individually dispersed (Fig. 7d). At day 7, the cells stretched along the surfaces of the WMC40 and WMC20 and extended to form cell layers (Fig. 7h,i), which were better than on WG scaffold (Fig. 7g).

### Proliferation and ALP activity of cells on scaffolds

**Figure 8:**
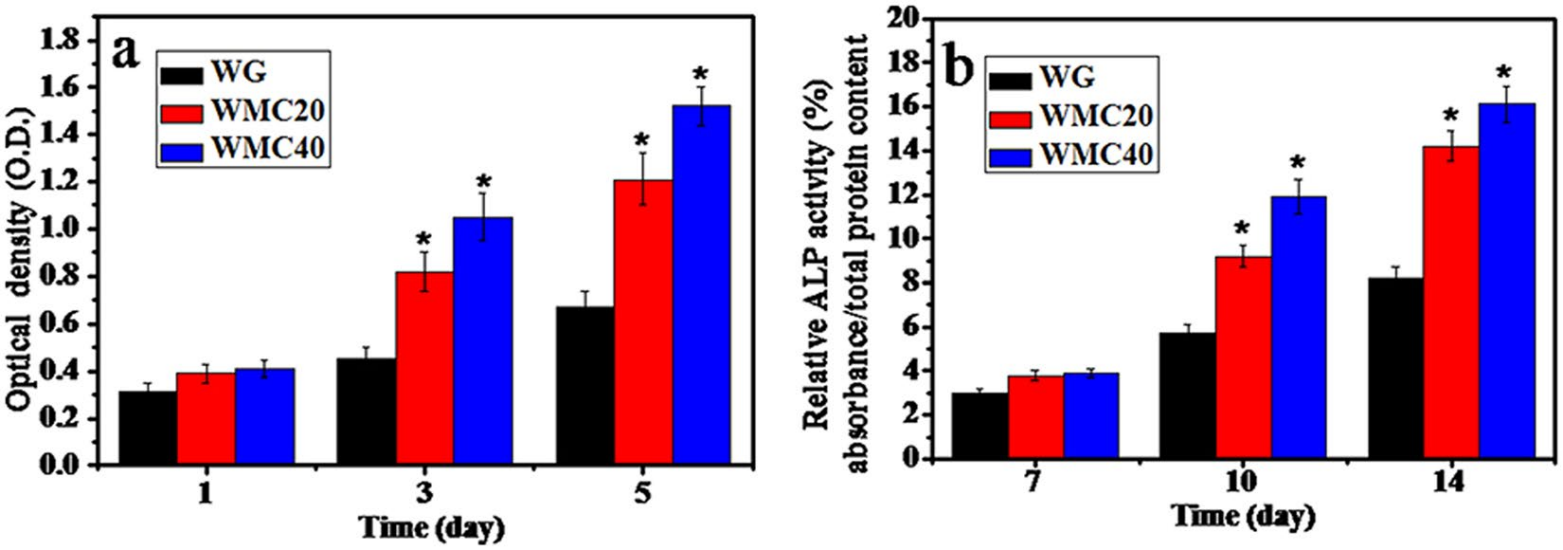
OD values (**a**) and ALP activity (**b**) of MC3T3-E1 cells cultured on the WG, WMC20 and WMC40 scaffolds with time (*p < 0.05).

Figure 8a shows the OD values of MC3T3-E1 cells cultured on the scaffolds at 1, 3 and 5 days. A significant increase of OD values was observed on WG, WMC20 and WMC40 with time, indicating all samples had good cytocompatibility. Obviously, the OD values for WMC20 and WMC40 were significantly higher than WG at 3 and 5 days, and WMC40 was significantly higher than WMC20, indicating that incorporation of m-MCS into WG significantly promoted the cell proliferation.

Figure 8b shows ALP activity of MC3T3-E1 cells after cultivating on WG, WMC20 and WMC40 for 7, 10 and 14 days. At day 7, no obvious difference was found for three kinds of scaffolds. However, at day 10 and 14, the ALP for WMC20 and WMC40 were significantly higher than WG, and WMC40 was significantly higher than WMC20, indicating that incorporation of m-MCS into WG significantly promoted the cell differentiation.

### Curcumin load/release from scaffolds

**Figure 9:**
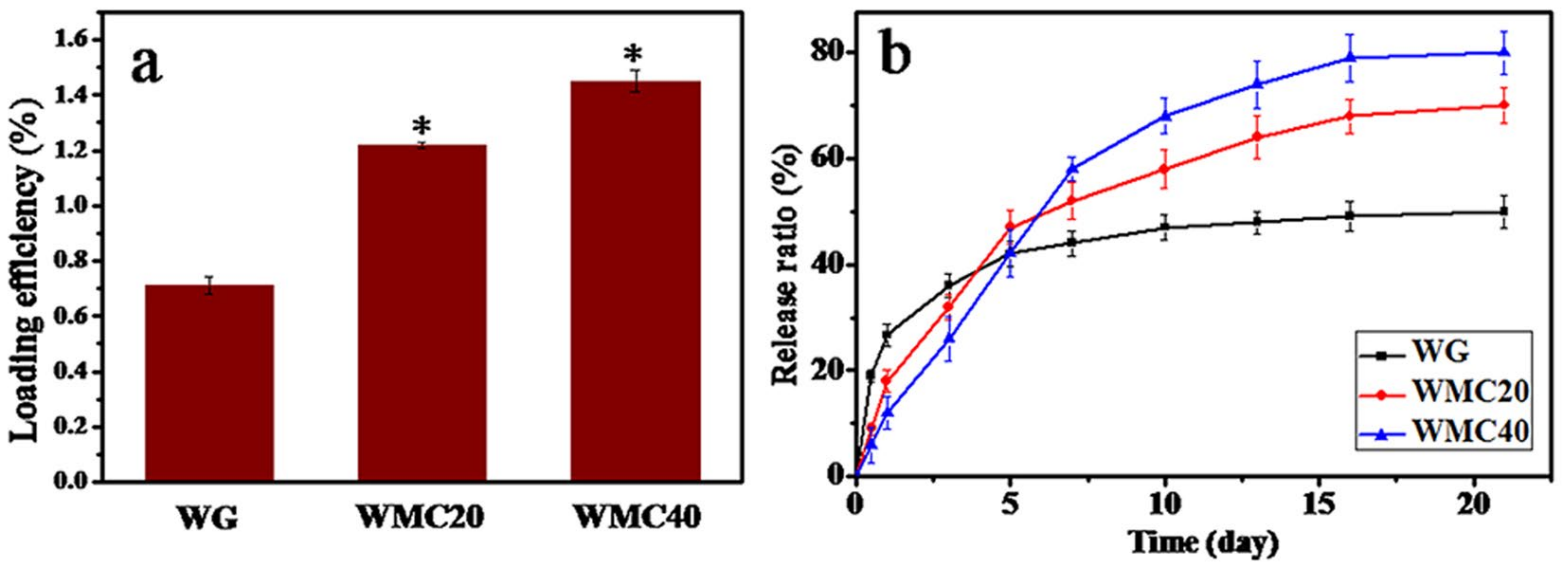
Curcumin loaded efficiency (**a**) on WG, WMC20 and WMC40 scaffolds, and cumulative release ratio (**b**) of curcumin from the scaffolds after immersion in PBS for 21 days.

Figure 9a shows the curcumin loaded efficiency of WG, WMC20 and WMC40 scaffolds. It was found that the efficiency of curcumin loaded into the scaffolds increased with m-MCS content (WG <WMC20 <WMC40). Figure 9b presents the cumulative release profiles of curcumin from the scaffolds. At day 1, the cumulative release of the curcumin from WG was 26.7% (an initial burst release) while WMC40 was 12.8% (an initial slow release). At day 21, the cumulative release of the curcumin from WG, WMC20 and WMC40 were 54.7%, 69.4% and 81.4%, respectively.

### Antibacterial of curcumin loaded scaffolds

**Figure 10:**
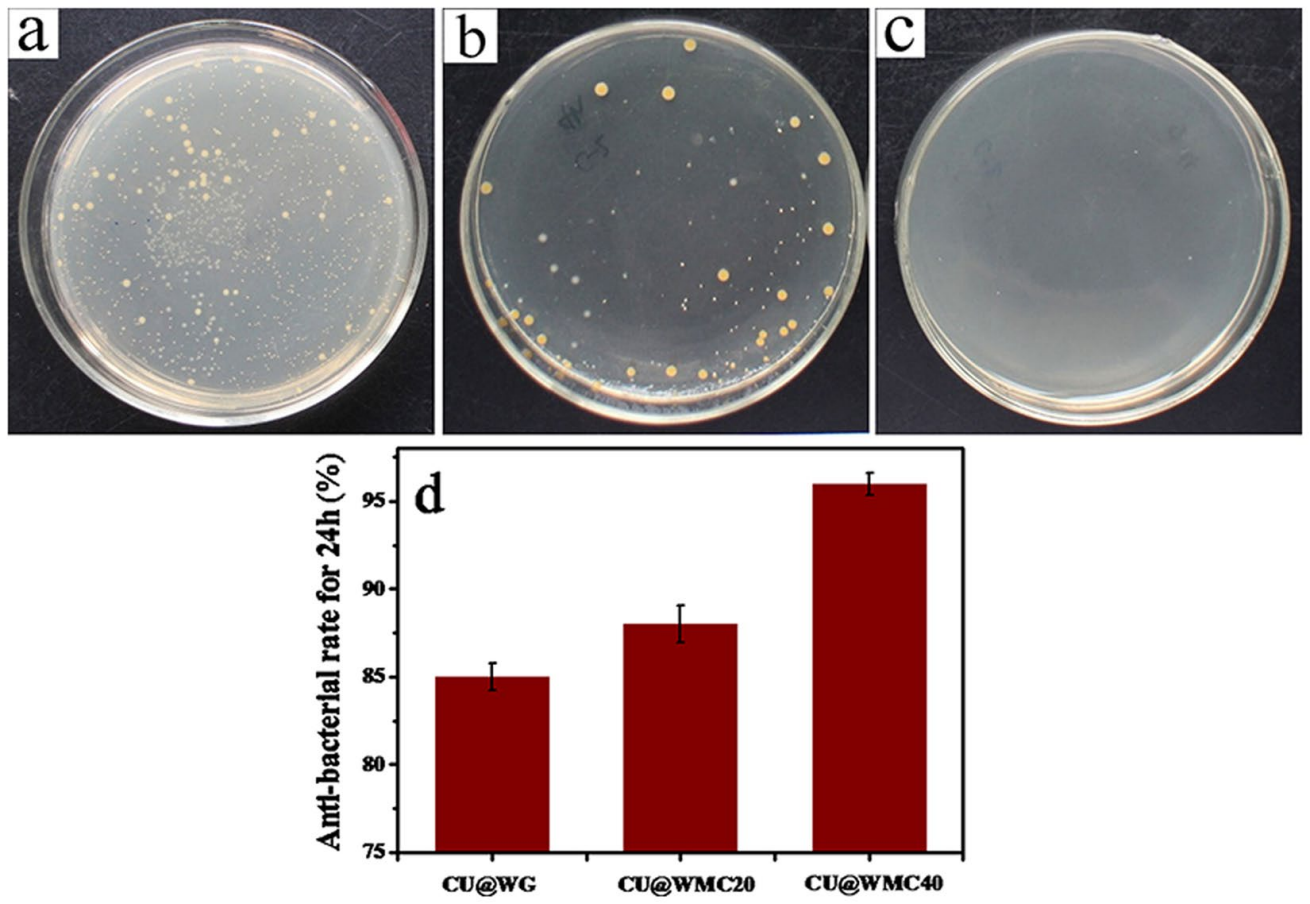
Distribution of *S. aureus* on CU@WG (**a**), CU@WMC20 (**b**) and CU@WMC40 (**c**) in culture dish at 24 hours, and antibacterial ratio (**d**) of CU@WG, CU@WMC20 and CU@WMC40 scaffolds at 24 hours.

Figure 10a–c shows the distribution of *Staphylococcus aureus*(*S. aureus*) for CU@WG, CU@WMC20 and CU@WMC40 in culture dish at 24 hours. No *S. aureus* was found on CU@WMC40 and a few *S. aureus* was found on CU@WMC20 while a large number of *S. aureus* on CU@WG (CU@WG < CU@WMC20 < CU@WMC40). Figure 10d shows the antibacterial ratio of CU@WG, CU@WMC20 and CU@WMC40 against *S. aureus*, it was found that the antibacterial ratios of CU@WG, CU@WMC20 and CU@WMC40 were 85%, 88% and 96%, respectively. The results indicated that the antibacterial properties of the scaffolds increased with increase of m-MCS content in the composites.

## Discussions

In this study, the macro-mesoporous scaffolds containing WG and m-MCS composites (WMC) was developed by incorporating m-MCS into wheat gliadin, which was crosslink with genipin. The results revealed that the both WG and WMC scaffolds had interconnected macropores with the size of about 500 μm, and the porosity the scaffolds obviously increased with the increase of m-MCS content (WG: 53.4%, WMC20:70.1% and WMC40:77.2%) because the WG scaffolds contained only macroporosity while WMC20 and WMC40 scaffolds contained not only macroporosity but also mesoporosity. In addition, the compressive strengths of the WG, WMC20 and WMC40 scaffolds were 1.93 MPa, 1.86 MPa, and 1.71 MPa, indicating that the increase of porosity of the scaffolds would not obviously decrease their mechanical strength. Moreover, the water absorption of the scaffolds was significantly increased with the increase of m-MCS content (WG: 474.2%, WMC20: 519.6% and WMC40:591.1%) because of the presence of mesoporosity in both WMC40 and WMC20 as compared with WG scaffolds without mesoporosity.

Excellent biomaterial should be gradually degradable *in vivo* during the course of new bone tissue formation and growth into scaffolds, which the materials were substituted by new bone tissue, and final bone defects were restored^20^. In this study, after soaking for 84 days, the degradation ratio of WG was 41.7 w% while WMC40 and WMC20 were 60.4 w% and 69.5 w%, respectively. The results indicated that the addition of m-MCS with large specific surface area/high pore volume into WG significantly enhanced the degradability of the composites scaffolds^8,11^. The presence of mesoporosity and improvement of the water absorption of WMC scaffolds caused by m-MCS might be beneficial to the degradation of the scaffolds. Moreover, the pH of the solution for WG decreased from 7.4 to 6.87 after 84-day immersion, indicating that some acidic degradable products (e.g amino acid) were emerged during soaking^21^. However, after 28-day immersion, the pH for WMC40 slightly increased from 7.40 to 7.59, and for WMC20 increased from 7.40 to 7.47, indicating that some basic products were emerged after the degradation of m-MCS, which might neutralize the acidic degradable products of WG. After 84-day immersion, the pH for WMC40 and WMC20 decreased to 7.23 and 7.10, respectively. The results indicated that with the increase of m-MCS content, the degradability of WMC scaffolds obviously enhanced, and the decrease of pH value could be slow down.

The ability of apatite formation, and even the quantity of the forming apatite, is very important for the binding of implant materials to living bone tissue, which can be evaluated in SBF solution *in vitro* ^22^. There are two main factors that affect the apatite mineralization process of bioactive materials in SBF, the availability of favorable nucleation sites and the localized super-saturation of ions concentrations^23^. In this study, the m-MCS in WMC scaffolds provided nucleation sites for apatite mineralization, and then subsequent crystal growth is thermodynamically driven as long as concentration gradient for the ions exists (such as Ca)^24^. After soaking in SBF for 7 days, the apatite formed on WMC40 scaffold surface, which would be attributed to the mineralization process of dissolution (m-MCS)-deposits (apatite)^25^. From the ICP analysis, the results revealed that the initial increases of concentration of Ca and Mg ions were due to the dissolution of m-MCS in the composites while the subsequent decrease of Ca and P ion concentration with the immersion time was attributed to the formation of apatite-layer on the composites surfaces^10,20^. Therefore, the results suggested that the WMC40 with improved bioactivity was expected to form a bone-bonding with host bone through the apatite-layer when implanted *in vivo* ^26^.

Mesoporous biomaterials have been reported not only to promote cell adhesion but also to enhance cell proliferation and differentiation^27^. Cell adhesion and spreading is an important step for the later cell proliferation and differentiation^28^. In this study, the results showed that the WMC scaffolds containing m-MCS strongly enhanced cell attachment, and with the increase of m-MCS content, more cell attachments were observed on the WMC40 scaffolds. Therefore, the presence of m-MCS in the WMC scaffolds played critical roles in the initial cell adhesion.

Previous studies confirmed that the dissolution products containing Si, Ca and Mg ions from bioactive glasses/ceramics stimulated osteoblast proliferation and differentiation^29^. Appropriate concentration of ions (such as Ca, Mg and Si ions) could promote osteoblasts activation by regulating the protein synthesis and ancillary processes, which could benefit osteoblasts proliferations and differentiation^30^. In this study, the proliferation of cells on the scaffolds obviously enhanced with the increase of m-MCS content, suggesting that the dissolution ions of m-MS from the WMC scaffolds improved cell proliferation.

In the process of osteogenic differentiation, ALP is an ectoenzyme involved in the degradation of inorganic pyrophosphate, which provide a sufficient local concentration of phosphate for mineralization^31^. In addition, ALP is expressed during the post-proliferative period of extracellular-matrix maturation, which has been widely recognized as a marker for osteoblast differentiation^32^. In this study, the results revealed that ALP activity of MC3T3-E1 cells on WMC40 scaffolds exhibited significantly higher levels of expression than those of others. Therefore, it can be suggested that the dissolution ions of m-MCS from the WMC scaffolds significantly promoted the cell differentiation.

Infection and its complications are one of the greatest threats to the health of orthopedic patients^33^. Open fracture, surgical infection and implant environment provided favorable conditions for bacterial adhesion and increased the probability of infection^34^. Biomaterial-related infections usually require extensive surgical debridement, implant extraction, and prolonged antibiotic treatment^35^. Therefore, resistance to bacterial infection would be a very desirable material performance for the biomaterials. In the study, the amount of CU loaded into WMC scaffolds increased with the increase of m-MCS content, and the WMC40 could load the highest amount of CU compared with WG scaffolds because WMC40 (with the highest porosity) contained not only macroporosity but also mesoporosity while WG (with the lowest porosity) had only macroporosity.

In addition, a burst release of CU from WG scaffolds at the early stage was observed because most of the CU might be absorbed on wall surface of macropores of the WG scaffolds. However, WMC40 scaffolds exhibited a slow release of CU, because most of the CU might be absorbed into mesopores of the WMC40 scaffolds. Therefore, it can be suggested that the mesoporosity in the WMC scaffolds played important roles for slow release of CU. Furthermore, the WMC40 containing the highest amount of CU exhibited good antibacterial activity *in vitro* against *S. aureus* as compared with WG scaffolds. The improvement of antibacterial effect of WMC40 was related to an increase of the CU content in the scaffolds with the highest porosity as compared with WG. Consequently, the addition of CU into WMC40 scaffolds was an effective method to develop the biocomposites with osteogenesis and antibacterial activity.

## Material and Methods

### Preparation and characterization of m-MCS/WG scaffolds

Mesoporous magnesium calcium silicate (m-MCS) was synthesized by the sol-gel technique as following: 6 g poly(ethylene glycol)-block-poly(propylene glycol) (P123, Sigma Aldrich Chemistry) was dissolved in 217 mL H_2_O and 36 mL ethanol until the solution was clarified. Then 17 mL HCl and 12.9 mL tetraethyl orthosilicate(TEOS) was added in the solution under stirring for 30 min, respectively, and temperature was kept at 38 °C. After that, 7.9 g Mg(NO_3_)_2_·6H_2_O, 7.3 g Ca(NO_3_)_2_·4 H_2_O and 30 mL H_2_O was added in the solution, and during this process the temperature was kept at 38 °C. After vigorous stirring (stirring rate of 50 rpm) in a magnetic stirrer (HJ-4, Guohua electric appliance co., Ltd, China) for 5 h, the milk-like solution was placed at 95 °C for 3 days. The as-prepared m-MCS were collected by pumping filtration, washed with deionized water, dried at 100 °C overnight, and sintered at 600 °C for 6 hours to remove the remaining structure-directing agent P123, and finally m-MCS was obtained. All the chemicals were obtained from Shanghai Lingfeng Chemical Regent co., Ltd. The inner microstructure of as-prepared m-MCS was characterized by transmission electron microscopy (TEM, JEM-2010, JEOL).

The wheat gliadin (WG) was purchased from Japan Tokyo Chemical Industry Co., Ltd. (TCI). The m-MCS/WG composite (WMC) scaffolds without m-MCS (WG), 20 w% m-MCS (WMC20) and 40 w% m-MCS (WMC40) content were prepared by genipin crosslinking and salt-leaching method. Briefly, m-MCS and WG powders were uniformly mixed in the certain percentage, and NaCl particles, which were sieved with diameter of 400–500 nm, were mixed into the compound after uniform stirring. The mixtures were consolidated in stainless steel molds (Φ12 × 2 mm) under a pressure of 2 MPa for 3 min. Then, the samples were placed in the 24-well plate with each sample occupying a single well, and 1 mL of genipin solution (10 mmol/L) was added into each well at 37 °C for 24 hours. Finally, WG, WMC20 and WMC40 scaffolds were obtained by soaking the samples in the deionized water for 36 hours (refreshing the deionized water every 12 hours) to remove NaCl, and vacuum freeze-drying at −55 °C for 24 hours. the WG, WMC20 and WMC40 scaffolds were characterized by scanning electron microscopy (SEM; S-3400N; Hitachi, Japan), Fourier transform infrared spectroscopy (FTIR, Thermo Nicolet 6700, Waltham, MA, USA) and X-ray diffraction (XRD) using the D/max 2550VB/PC diffractometer (Rigaku, Tokyo, Japan).

### Porosity, water absorption and compressive strength of WMC scaffolds

The porosity of the WG, WMC20 and WMC40 scaffolds (P) was measured by applying the Archimedes principle^36^. A density bottle was used as the displacement liquid to measure the porosity of the scaffolds. Ethanol (density ρ_0_) was used as the displacement liquid and operated at 30 °C. The calculation of P was performed by the following equation (1):

1 $${\rm{P}}=\frac{{W}_{2}-{W}_{3}-{W}_{s}}{{W}_{1}-{W}_{3}}\times 100 \% $$

W_1_ = the weight of a density bottle filled with ethanol. Ws = the weight of the scaffold. W_2_ = the weight of the density bottle supplemented with ethanol to full after the scaffold immersed into it. W_3_ = the weight of the density bottle after taking off the scaffold.

The WG, WMC20 and WMC40 scaffolds were incubated in de-ionized water, and the hydrated samples were removed from water at intervals of 1, 5, 10, 15, 30, 60 and 120 min. Removal of excess superficial water with filter paper was carried out and the samples weights were recorded. The water absorption ratio was calculated by the following equation (2):

2 $$\mathrm{Water}\,\,{\rm{absorption}}\,{\rm{ratio}}=\frac{{W}_{H}-{W}_{D}}{{W}_{D}}\times 100 \% $$

where W_H_ = soaked weight, W_D_ = dry weight of the scaffolds.

The compressive strengths of the WG, WMC20 and WMC40 scaffolds (size of 10 × 10 × 10 mm) were measured by a universal testing machine (AG-2000A, Shimadzu, Japan) at a constant loading rate of 1 mm·min^−1^.

### *In vitro* degradability of WMC scaffolds

The WG, WMC20 and WMC40 scaffolds were immersion in Tris [hydroxymethyl] aminomethane-HCl (Tris-HCl) solution (pH = 7.4) at 37 °C for 84 days (the solid/liquid mass ratio was 0.5 g/g), and the Tris-HCl solution was refreshed once a week^37^. The scaffolds were removed from Tris-HCl solution at different time points (1, 3, 7, 14, 21, 28, 35, 42, 56, 70, 84 days), and dried at 50 °Cin an oven overnight. The percentage of weight loss was determined as follows equation (3):

3 $${\rm{weight}}\,{\rm{loss}}\,( \% )=\frac{{W}_{0}-{W}_{t}}{{W}_{0}}\times 100$$

Where W_0_ was the initial weight and W_t_ represented the dry weight at time t.

The pH change of the solution was measured at different time points (1, 3, 7, 14, 21, 28, 35, 42, 56, 70, 84 days) after the scaffolds soaking. For pH measurements, Tris-HCl solution was not refreshed during 84-day of immersion.

### *In vitro* mineralization of WMC scaffolds

*In vitro* bioactivities of the scaffolds were assessed by apatite mineralization ability in simulated body fluid (SBF, pH = 7.4) at 37 °C for 7 days, and SBF was prepared by dissolving NaCl, NaHCO_3_, KCl, K_2_HPO_4_·3H_2_O, MgCl_2_·6H_2_O, HCl (1 mol/L), CaCl_2_, Na_2_SO_4_ and NH_2_CH_2_OH^38^. The scaffold (Φ12 × 2 mm) were soaked in SBF, and the solution volume/the specimen weight ratio was 20 ml·g^−1^. After soaking, the scaffolds were removed from SBF, and washed by deionized water and then dried in an oven at 50 °C for 24 h. The surface morphology after immersion in SBF was determined by SEM, and the surface composition and functional groups of deposits were determined by energy dispersive spectroscopy (EDS; Falcon, USA) and FTIR. The ion concentrations (Si, Mg, Ca and P ions) in the SBF were determined by inductively coupled plasma atomic emission spectroscopy (ICP-AES; IRIS 1000; Thermo Elemental, USA) after the WMC40 soaking into SBF at 1, 3, 5, 7 and 14 days.

### Cells response to WMC scaffolds

#### Cell morphology

The WG, WMC20 and WMC40 scaffolds were placed in a 24-well plate, which sterilized in 100% ethanol for 1 hour and then UV radiated for 1 hour. The MC3T3-E1 cells, which were purchased from Shanghai Institutes for Biological Science, Chinese Academy of Science (Shanghai, China), were seeded on these substrates (scaffolds) at a density of 2 × 10^4^ cells/well and incubated at 37 °C in the atmosphere of 5% CO_2_ and 95% air for 1, 3 and 5 days. The specimens were washed 3 times with phosphate buffered saline (PBS), and cells on the surfaces of the samples were fixed with 4% glutaraldehyde for 2 h. Finally, the cell-seeded samples were washed with PBS followed by sequential dehydration in graded ethanol (30, 50, 70, 90 and 100%). The cell morphology adhered on the specimens was observed by a scanning electron microscope (SEM, S-3400N, Hitachi High-Technologies Co., Tokyo, Japan) at 5 kV of accelerating voltage after the fixation and dehydration followed by gold–palladium coating (magnetron sputtering machine; MSP-1S, Vacuum Device Co., Ibaraki, Japan). The forth to sixth passage cells were used for experiments.

#### Cell proliferation

Cell proliferation was determined after 1, 3 and 5 days of incubation using methyl thiazolyl tetrazolium (MTT) assay. Briefly, the samples (WG, WMC20 and WMC40) were placed in a 24-well plate. 1 mL of cell suspension at a cell density of 5 × 10^3^ viable cells was seeded on the samples and then kept at 37 °C in a humidified incubator with 5% CO_2_. MTT reagent (3-[4,5-dimetylthiazole-2-yl]- 2,5-diphenyltetrazolium bromide), which was enzymatically converted by living cells into a blue/purple formazan product, was added to each sample and incubated at 37 °C for 4 h. The blue formazan product was solubilized with dimethylsulfoxide, and the liquid portion of each sample was removed for the assay, which was performed in a 96-well plate. Absorbance was read using a microplate reader (MTP-32 Microplate Reader, Corona elect, Ibaragi, Japan) at 570 nm. The OD values at day 3 and 5 were normalized to those at day 1 because the numbers of attached cells grown on different samples were different at day 1.

#### Alkaline phosphatase activity

MT3T3-E1 cells were seeded on the scaffolds (WG, WMC20 and WMC40), and then incubated with the osteogenic induction medium in 24-well plates. At day 7, 10 and 14, 200 μL 1% Nonidet P-40 (NP-40) solution was added into each well after the culture medium aspirated, and incubated for 1 h. The cell lysate was obtained and centrifuged. Exact 50 mL of supernatant was added to 96-well plates, 50 μL of 2 mg/mL p-nitrophenyl-phosphate (Sangon, Shanghai, China) substrate solution composed of 0.1 mol L^−1^ glycine, 1 mmol L^−1^ MgCl_2_·6H_2_O was added and incubated for 30 min at 37 °C. The reaction was quenching by adding 100 μL of 0.1 N NaOH, and the absorbance of ALP was quantified at a wavelength of 405 nm using a microplate reader (SPECTRAmax 384, Molecular Devices, USA). ALP activity was normalized to protein quantity of scaffolds. ALP activity was normalized to protein quantity of scaffolds, and all the experiments were performed for 3 times.

### Curcumin load/release of WMC scaffolds

To investigate the effects of m-MCS on curcumin load/release properties of WMC scaffolds, curcumin (CU) was dissolved in 30% ethanol PBS (pH = 7.2) with a concentration of 300 μg/mL. The scaffolds (WG, WMC20, WMC40) were added into CU solution (scaffold/CU solution = 5 g/50 mL) with stirring for about 24 hours.

The amount of curcumin before and after stirring was analyzed by high-performance liquid chromatography (HPLC) according to the modified method of Wichitnithad *et al*.^39^. An Agilent HPLC system (Agilent 1100, USA) equipped with ultraviolet (UV) detector (425 nm) and a column (ODS2 C18 200 × 4.6 mm) was used. The loading efficiency of scaffolds were calculated by the following equation (4):

4 $${\rm{LE}}={\rm{Wb}}-\mathrm{Wa}/\mathrm{Wb}\times \mathrm{100} \% $$

Wa: the amount of curcumin in solution before the scaffolds were added. Wb:The amount of curcumin in solution after the scaffolds were added for 24 h.

The scaffolds were placed into the plastic bottles with 2 mL 30% ethanol PBS (pH = 7.2) solution. The PBS solution (200 μL) was taken out at each time point (0.5, 1, 3, 5, 7, 10, 13, 16, 21 days) to test the concentration of CU by enzyme-linked immunosorbent assy (ELISA, SPECTRAmax 384, Molecular Devices, USA) at the wavelength of 426 nm, and the fresh PBS solution (200 μL) was added back to the plastic bottles. For testing the CU-release properties from the scaffolds, the standard curve with certain concentrations of CUR was measured in order to make a regression equation. The concentration and release ratio was calculated by regression equation.

### Antibacterial property of WMC scaffolds

The antibacterial ability of the scaffolds was assessed by the bacteria counting method using *Staphylococcus aureus* (*S. aureus*, ATCC 25923). All the scaffolds were sterilized by ultraviolet light overnight. A 60 mL suspension containing 10^7^ CFU/mL of the bacteria was dropped onto the scaffolds. The scaffolds with the bacteria suspension were incubated at 37 °C for 24 h and then put into the sterilized centrifugal tube containing 4 mL of physiological saline. The tube was agitated vigorously for 30 s using a vortex mixer to dissociate bacteria from the scaffolds. Afterwards, the dissociated bacteria suspension was diluted 10, 100, and 1000 times with sterile physiological saline. 100 mL of the diluted bacteria suspension were introduced to the standard tryptic soy broth (TSB) agars and incubated at 37 °C for another 16 h. The active bacteria were counted according to the National Standard of China GB/T 4789.2 protocol. The antibacterial ratio was calculated using the following equation (5):

5 $$({\rm{C}}-{\rm{T}})/T\times 100 \% $$

where C is the average number of the bacteria colonies on the samples (CFU/sample) and T is the average number of bacterial colonies on the testing samples (CFU/sample).

### Statistical analysis

A minimum of five samples was tested per group for physical and chemical properties, and six parallel samples per group for the cell experiments. p < 0.05 was considered statistically significant. All quantitative data were analyzed with Origin pro8.0 and expressed as the mean ± standard deviation (M ± s.d.).

## Conclusion

Bioactive macro-mesoporous scaffolds based on m-MCS/WG composites crosslinking with genipin was developed. Compared with the macroporous WG scaffolds, WMC20 and WMC40 scaffolds with m-MCS not only contained macroporosity but also mesoporosity. The increasing amount of m-MCS significantly improved the porosity (mesoporosity), water absorption of WMC scaffolds while slightly reduced the compressive strength. The incorporation of m-MCS into WG obviously enhanced degradability and apatite mineralization of WMC20 and WMC40 compared to WG scaffolds. In addition, the WMC scaffolds containing m-MCS with mesoporosity stimulated the attachment, proliferation and differentiation of MC3T3-E1 cells. Furthermore, WMC scaffolds with mesoporosity could load more CU and revealed sustained release of CU. In conclusion, the incorporation of m-MCS into WG was a useful approach to obtain biocomposite scaffolds with improved properties, which might be used as bone scaffolds for repair bone defects and anti-infection.

## Electronic supplementary material

Supplementary Information for TEM image of m-MCS, Compressive stress-strain curve of different samples and FTIR analysis of deposits on WMC40 surface after soaking in SBF for 7 days
