# Supplementary material for: Influences of mesoporous magnesium calcium silicate on mineralization, degradability, cell responses, curcumin release from macro-mesoporous scaffolds of gliadin based biocomposites
Source: Sci Rep. 2018 Jan 9;8:174. doi: 10.1038/s41598-017-18660-9 (PMC5760662; doi:10.1038/s41598-017-18660-9)
Supplement: Supplementary file 1 — Supplementary Information for TEM image of m-MCS, Compressive stress-strain curve of different samples and FTIR analysis of deposits on WMC40 surface after soaking in SBF for 7 days [file 41598_2017_18660_MOESM1_ESM.doc]

**Influences of mesoporous magnesium calcium silicate on mineralization, degradability, cell responses, curcumin release from macro-mesoporous scaffolds of gliadin based biocomposites**

Sicheng Wang,^a,b^ Zhengrong Gu^a,c#^, Zhiwei Wang^a^, Xiao Chen^a^, Liehu Cao^a^, Liang Cai^d^, Qu Li^a^, Jie Wei^d^, Jung-Woog Shin^e^, Jiacan Su^a^*


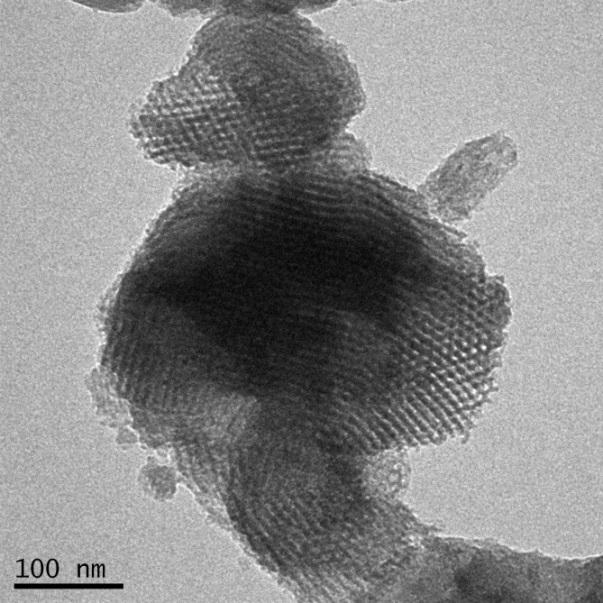


Supplementary Figure 1. TEM image of m-MCS


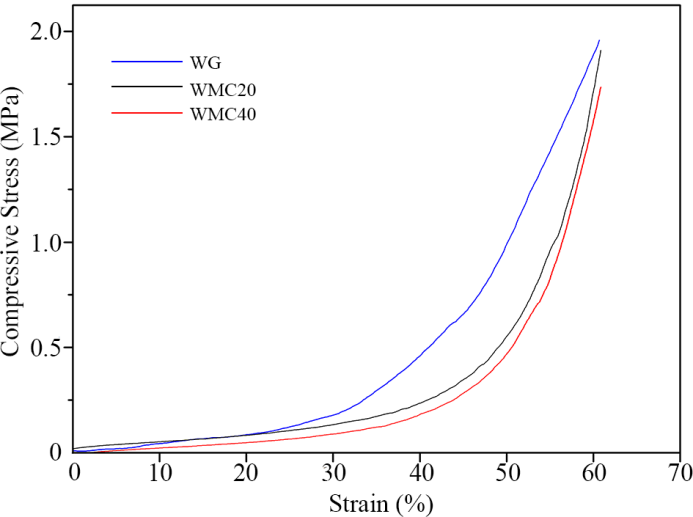


Supplementary Figure 2. Compressive stress-strain curve of different samples


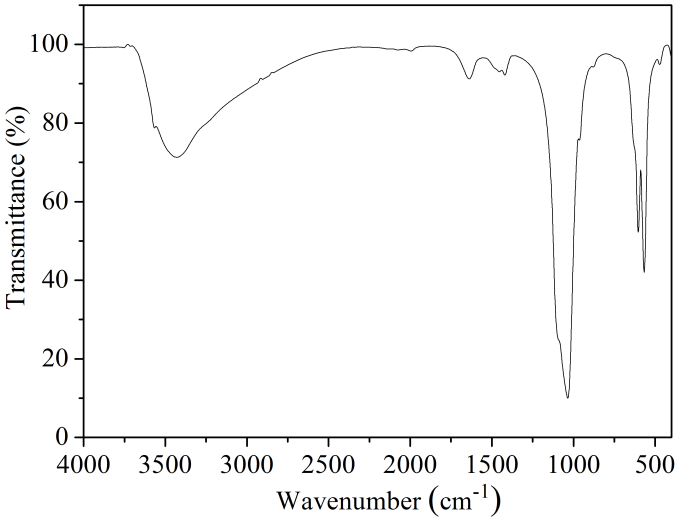


Supplementary Figure 3. FTIR analysis of deposits on WMC40 surface after soaking in SBF for 7 days
